# Molecular drivers of osteogenesis imperfecta: a cellular and extracellular collagen disease

**DOI:** 10.1042/CS20255642

**Published:** 2025-12-18

**Authors:** Silvia Cotti, Wendy Pérez Franco, Antonella Forlino

**Affiliations:** 1 Department of Molecular Medicine, Biochemistry Unit, University of Pavia, Pavia, Italy

**Keywords:** bone, collagen, heritable connective tissue disease, osteogenesis, osteogenesis imperfecta, post-translational modifications, rare bone disorders

## Abstract

The clinical hallmarks of osteogenesis imperfecta (OI), often referred to as ‘brittle-bone disease’, are bone fragility and skeletal deformities that are usually accompanied by extra skeletal manifestations. OI is a family of collagen I-related disorders, currently classified into 23 distinct types and 5 OI-like forms, with variable phenotypic severity ranging from mild to lethal. At the molecular level, the pathophysiology of OI is driven by alterations in collagen I structure, primarily caused by dominant mutations in collagen genes (affecting approximately 85% of patients). It can also result from dominant, recessive, or X-linked defects in proteins involved in collagen biosynthesis, extracellular matrix organization, mineralization, or bone forming cell differentiation and/or activity. This review illustrates the different OI forms from a collagen I perspective, its complex biosynthetic process is first described, followed by a classification of the OI and OI-like causative mutations grouped based on whether the resulting collagen molecule is overmodified, undermodified, or unaltered. The underlying molecular mechanisms and the consequences at cellular and extracellular levels leading to the OI phenotype are discussed. An overview is provided on how newly discovered molecular pathways altered in OI can guide the development of innovative therapies aiming at increasing bone mass and improving bone quality in OI patients.

## Introduction

Collagen I is the predominant fibrillar collagen in vertebrates and it is an essential component of the extracellular matrix (ECM) in several tissues [1,2]. It is the most abundant protein in bone, where it constitutes over 90% of the organic matrix, and it plays a crucial role in the mineralization process [3]. Collagen I is a heterotrimer composed of two α1(I) and one α2(I) chains, encoded by *COL1A1* and *COL1A2* genes, respectively. Translation occurs in the endoplasmic reticulum (ER), where two proα1(I) and one proα2(I) chains are assembled from the C- to the N-terminal end. The resulting structure consists of a triple-helical region including 338 Gly-Xaa-Yaa triplet repeats, flanked by a globular amino-terminal domain (N-propeptide) connected via a short linear N-telopeptide and a globular carboxyl-terminal domain (C-propeptide) linked by a C-telopeptide sequence [4].

The rate-limiting phase in the procollagen folding is the *cis-trans* isomerization of prolyl peptide bonds of the triple helical domain, a process catalyzed by peptidyl-prolyl *cis-trans* isomerases (PPIases) [4,5]. A glycine residue at every third position is necessary, being the only amino acid with a small side chain fitting the inner space of the helix, while the other two amino acids of the triplet are often proline and 4-hydroxyproline (Hyp) [6,7]. ER chaperones, such as protein disulfide isomerase (PDI) and immunoglobulin heavy-chain-binding protein (BiP), interact with procollagen molecules to prevent the secretion of unassembled procollagens from the ER [8,9].

Specific and unique post-translational modifications (PTMs) occur within the ER before the proα chain assembly and folding. One of the most critical modifications is the hydroxylation of proline and lysine residues catalyzed by the enzymes prolyl-4-hydroxylase (P4H1) and lysyl hydroxylase 1 (LH1) and 2 (LH2), respectively [10]. P4H1 hydroxylates in position C4 almost every proline residue at the Yaa position, ensuring inter-chain hydrogen bonds relevant for folding and stability of the triple helical domain [10]. LH1 and LH2 hydroxylate lysine residues in the triple helical and N-/C- telopeptide domains, respectively. Following hydroxylation, certain hydroxylysine (Hyl) residues undergo an unusual ER glycosylation where galactose or galactose/glucose mono- and disaccharides are covalently linked by the enzymes hydroxylysyl-galactosyltransferase and galactosyl-hydroxylysyl-glucosyltransferase, respectively. This glycosylation is necessary for proper fibril formation and ECM organization [11].

Prolyl 3-hydroxylase 1 (P3H1) complexes with cartilage-associated protein (CRTAP) and cyclophilin B (CyPB) to hydroxylate specific proline sites in α1(I) and α2(I) [12]. 3-Hyp residues are essential for stabilizing the collagen triple helix, as they increase the thermal stability of the collagen molecule and also modulate collagen fibril assembly in the extracellular space [13,14].

The chaperone heat shock protein 47 (HSP47) interacts with folded procollagen and prevents local triple helical unfolding and intracellular aggregation [15,16]. Furthermore, HSP47 interacts with the ER transmembrane protein transport and Golgi organization 1 (TANGO1) favoring procollagen I exit from the ER through coat protein complex II (COPII) vesicles/ tunnels [17]. In the secretory path, lysine hydroxylation and Hyl glycosylation can occur mediated by lysyl hydroxylase 3 (LH3) [18–21]. Finally, procollagen is secreted from *trans* Golgi vacuoles into the extracellular environment [22–24].

In the pericellular space, the globular N- and C- propeptides are removed by zinc-proteases ADAM metallopeptidase domain 2 (ADAM2) and bone morphogenic protein 1 (BMP1), respectively. The triple helical collagen I molecules self-assemble into highly organized fibrils, which are stabilized by covalent cross-links mediated by the enzyme lysyl oxidase, and the fibrils grow by addition of individual collagen molecules to the ends of the fibrils or by end-to-end fusion with nascent short fibrils [25–27]. Mutations in collagen chain amino acid composition and defects in proteins/enzymes involved in collagen I synthesis are often responsible for altered collagen structure associated with either excessive or limited post-translational modifications as well as for reduced amount of protein assembled in the ECM.

Osteogenesis imperfecta (OI), a family of collagen I-related disorders, is characterized by skeletal deformity and fragility due to both bone ECM microarchitecture disruption [28] and altered bone-forming cells homeostasis [29]. OI is a rare disease with an estimated incidence of 1 in 15–20,000 live births [30] and generally occurs with equal frequency among males and females and racial and ethnic groups (https://www.genome.gov/Genetic-Disorders/Osteogenesis-Imperfecta. Accessed January 24^th^ 2025).

Approximately 85% of OI cases are caused by dominant mutations in collagen I genes [31,32]. These mutations were the first to be identified as causative for the disease and are, therefore, referred to as responsible for the classical forms of OI, classified by Sillence into: mild (type I), lethal (type II), severe (type III), and moderate (type IV) [33].

The remaining 15% of OI cases result from mutations in genes encoding proteins that are crucial for collagen I biosynthesis, folding, and assembly, as well as proteins that are essential for bone mineralization and osteoblast differentiation and/or activity [34]. Clinical manifestations in these non-classical OI can also range from mild to lethal [30]. Based on a genetic classification, the newly identified forms were named with consecutive numbers that reach now XXIII in the OMIM database, although a functional classification was also proposed [35,36]. Except for mutations in interferon-induced transmembrane protein 5 (*IFITM5*) that are autosomal dominant and in membrane-bound transcription factor peptidase site 2 (*MBTPS2*) that are recessive X-linked, all the others have autosomal recessive transmission [30].

While research on OI has predominantly focused on the skeletal manifestations, which is why it is also known as ‘brittle bone disease’, the disorder is associated with several extra-skeletal complications, including blue sclerae, dentinogenesis imperfecta (DI), cardiovascular and respiratory dysfunction, hearing loss, and joint hypermobility [37,38].

In OI patients, the structure and function of growth plate cartilage is disrupted, impairing endochondral ossification and leading to reduced bone growth and short stature [39]. Moreover, extraskeletal tissues containing a substantial amount of collagen I are compromised in OI, and the presence of defective collagen contributes significantly to their deterioration. In recent years, research has increasingly focused on these tissues, highlighting how their decline is often accompanied and exacerbated by aging processes. The main described extraskeletal OI features, namely blue-gray sclera, hearing loss, and dental abnormalities are mostly associated with *COL1A1/COL1A2* mutant variants, but not exclusively [37,40]. Others, such as muscle weakness, cardiovascular, and pulmonary complications appear in both collagenous and non-collagenous OI mutations [37]. Prevalence of valvular disease, heart failure, and atrial fibrillation is higher in OI patients than healthy individuals, and they appear in all types of OI and all ages [41]. Of note, cardiovascular complications were found more pronounced in pediatric patients carrying *COL1A1* mutations than in those with *COL1A2* or non-collagen gene mutations [42]. Respiratory dysfunction in OI is predominantly attributed to scoliosis and rib fractures; however, decreased tidal volume and forced vital capacity can also occur in patients without these skeletal deformities [43]. This respiratory compromise is more pronounced in individuals with severe OI compared to those with moderate OI forms [44]. Finally, neurological manifestations have also been described due to abnormalities in the cranial structure leading to underlying brain malformations, aneurysms, basilar invaginations, among others in both humans and animal models [45,46].

Molecular mechanisms behind the heterogeneity found in OI patients are still puzzling the field, and until now, it is not possible to accurately predict the phenotype for a given either dominant or recessive or X-linked mutation. In general, quantitative collagen I defects lead to milder clinical manifestations, while qualitative mutations result in phenotypes ranging from mild to lethal [31]. The recessive OI forms often present moderate to severe/lethal phenotypes, sometimes indistinguishable from the severe dominant forms (e.g. OI type III/II), but an accurate genotype-phenotype prediction is challenging. Recessive mutations causing loss of function in proteins involved in collagen I post-translational modification produce the most severe forms, a severity partially attributed to the role these proteins also play in the processing of other types of collagen [47–49].

Several attempts have been made to identify useful biomarkers for predicting OI severity and/or progression. A quantitative proteomic study using OI human fibroblasts carrying *COL1A1* or *COL1A2* defects showed a differential expression of 17 proteins in lethal and severe OI. Some of them (e.g. decorin, fibrillin-1, nestin, and paladin) directly correlate to the severity of the disease [50]. A recent human study also demonstrated that levels of C-type natriuretic peptide (CNP) and its biologically inactive fragment, both established biomarkers of growth, are reduced in OI in a manner that correlates with phenotypic severity [51]. Moreover, the differential dysregulation of micro-RiboNucleic Acids may contribute to the altered regulation of genes implicated in OI pathophysiology [52]. In this review, taking into account both genetic and functional classification, we grouped the causative mutations for classical and more recently described OI and OI-like forms focusing on the presence of excessive, reduced, or normal collagen post-translational modifications (Table 1). The underlying molecular mechanisms and the consequences at cellular and extracellular levels are discussed. We also explore how the newly discovered altered signaling pathway can guide the development of targeted therapies based on the specific biochemical profile, supporting the advancement of innovative clinical approaches.

**Table 1:** OI-causing genes and the effects on collagen I posttranslational modification

Collagen I structure	Impaired biochemical pathway	Intracellular collagen retention	Gene	Protein	OI type (OMIM number)	Main clinical features	Phenotype severity
OVERMODIFIED	Collagen synthesis	Intracellular collagen retention and delayed secretion (based on patient fibroblasts and murine osteoblasts)	*COL1A1* *COL1A2*	proα1(I) proα2(I)	II (166210) III (259420) IV (166220)	Multiple fractures Scoliosis DI Blue or white sclerae	Lethal, severe to moderate
		Intracellular collagen retention and delayed secretion (based on patient fibroblasts and murine osteoblasts)	*CRTAP*	CRTAP	VII (610682)	Multiple fractures Scoliosis Blue or white sclerae Rhizomelia	Lethal to severe
		Intracellular collagen retention and delayed secretion (based on patient fibroblasts and murine osteoblasts)	*P3H1*	P3H1	VIII (610915)	Multiple fractures Scoliosis Blue or white sclerae Rhizomelia Unusual dental abnormalities	Lethal to severe
		*Intracellular collagen retention and delayed secretion (based on patient fibroblasts and murine osteoblasts)	*PPIB*	CyPB	IX (259440)	Multiple fractures Scoliosis Blue or white sclerae No rhizomelia	Lethal to moderate
		Intracellular collagen retention and delayed secretion (based on patient fibroblasts)	*SPARC*	Osteonectin	XVII (616507)	Multiple fractures Scoliosis White sclerae DI in one patient Delayed motor development	Severe progressive
UNDERMODIFIED	Calcium homeostasis; Cell adhesion	Intracellular collagen retention (based on patient fibroblasts)	*TMEM38B*	TRIC-B	XIV (615066)	Multiple fractures Scoliosis White sclerae	Asymptomatic to severe
NORMAL	Collagen synthesis	No intracellular collagen I aggregation (based on patient fibroblasts)	*COL1A1* *COL1A2*	proα1(I) proα2(I)	I (166200)	Multiple fractures Scoliosis DI Blue sclerae	Mild
		Intracellular collagen I retention (based on patient fibroblasts)	*P4HB*	PDI	OI-Cole Carpenter Syndrome type I	Frequent fractures Ocular proptosis Hydrocephalus	Mild to severe
	Collagen folding and cross-linking	Intracellular collagen I retention and delayed secretion (demonstrated in patient and murine fibroblasts)	*SERPINH1*	HSP47	X (613848)	Multiple fractures Scoliosis DI in one patient Blue or white sclerae	Lethal to severe
		No to Mild intracellular collagen I aggregation associated with underhydroxylation of the lysine residues in telopeptides (based on patient fibroblasts)	*FKBP10*	FKBP65	XI (610968)	Recurrent fractures Kyphoscoliosis DI except in one patient White or gray sclerae Fish scale-like lamellae	Moderate
		Underhydroxylation of the lysine residues in telopeptides	*PLOD2*	LH2	Bruck type 2 syndrome	Fractures Osteoporosis Joint contractures Low levels of collagen cross-links degradation products in urine	Severe progressive
	Collagen trafficking	Intracellular collagen retention and delayed secretion (based on patient fibroblasts)	*KDELR2*	KDELR2	XXI (619131)	Multiple fractures Scoliosis in one patient No DI except one patient Normal or blue sclerae	Severe progressive
		Accumulation of procollagen I in the ER (demonstrated in murine osteoblasts)	*CREB3L1*	OASIS	XVI (616229)	Multiple fractures No DI Blue sclerae	Lethal to mild
		Significantly reduced collagen I secretion (in patient fibroblasts)	*MBTPS2*	S2P	XIX (301014)	Fractures Variable scoliosis Blue or white sclerae	Moderate
		Accumulation of collagen in ER (in patient fibroblasts)	*SEC24D*	SEC24D	Cole-Carpenter syndrome type 2	Pre and postnatal fractures Craniofacial deformities Osteopenia Blue-gray sclerae in one patient	Severe
	Collagen cleavage	Decreased expression and secretion of collagen I (based on patients fibroblasts)	*BMP1*	BMP1	XIII (614856)	Recurrent fractures Kyphoscoliosis in one patient No DI White (blue in one patient) sclerae	Mild to severe
	Osteoblast differentiation and function	Not reported	*SP7*	OSTERIX	XII (613849)	Recurrent fractures No DI but delayed tooth eruption Normal sclerae	Moderate
		Not reported	*WNT1*	WNT1	XV (615220)	Recurrent fractures No DI Blue sclerae in some individuals Neurological manifestations	Moderate
		Not reported	*LRP5*	LRP5	Osteoporosis	Osteoporosis	Moderate
		Intracellular collagen I aggregates (in patient fibroblasts)	*MESD*	MESD	XX (618644)	Fractures No DI, but oligodontia and disorganized dentition White or blue sclerae	Lethal to severe
	Mineralization	Decreased expression and secretion of collagen I (in patient osteoblasts)	*IFITM5*	BRIL	V (610967)	Frequent fractures Scoliosis in one patient DI in one patient only White or gray-blue sclerae Hyperplastic callus Mesh-like lamellae organization	Moderate (MALEP-BRIL) Severe progressive (BRILp.S42L)
		Normal collagen I secretion (from patient fibroblasts)	*SERPINF1*	PEDF	VI (613982)	Frequent fractures No DI White, blue, or gray sclerae ‘Fish-scale' bone lamellae Excessive osteoid	Severe progressive
		Normal expression and modification of collagen I	*PLS3*	PLS3	Osteoporosis (300910, 306950)	Osteoporosis Recurrent fractures Rare extraskeletal OI manifestations	Moderate to severe, in male is more severe being X-linked
	mRNA regulation	Quantitative and qualitative defects in collagen I production (from murine osteoblasts)	*TENT5A*	FAM46A	XVIII (617952)	Fractures DI Blue sclerae	Lethal to severe
	Cellular signaling	Significant reduction of collagen I protein (in patient osteoblasts)	*CCDC134*	CCDC134	XXII (619795)	Multiple fractures Scoliosis No DI White or gray or blue sclerae	Severe
	Cellular signaling Collagen synthesis	Not reported	*PHLDB1*	PHLDB1	XXIII (620639)	Recurrent fractures No DI Blue sclerae	Mild

One mutation reported in *PPIB did not cause delayed collagen folding and patients presented normal collagen I although the molecular mechanism remain poorly understood.

## OI forms characterized by overmodified collagen

### Glycine substitutions, splice variants, insertions, and deletions in *COL1A1*/*COL1A2*

**Figure 1:**
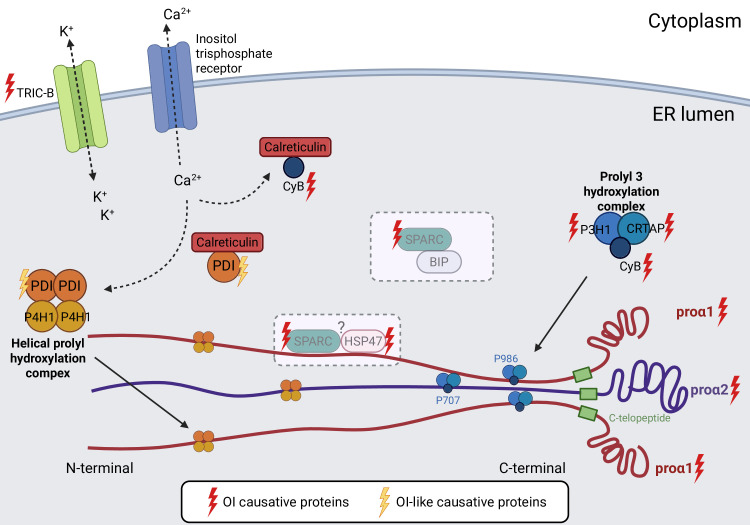
Proteins driving aberrant post-translational modifications of collagen I in OI. Mutations in collagen I chains (proα1(I) and proα2(I), not reported in the figure), as well as defects in the Prolyl 3 hydroxylation complex constituted by prolyl 3-hydroxylase (P3H1), cartilage-associated protein (CRTAP)*,* and cyclophilin B (CypB) delay the proper folding of procollagen I extending PTMs exposure time and causing triple helix overmodification. In contrast, mutations in TRIC-B, an ER trimeric intracellular cation channel type B, impair intracellular calcium flux that has an effect on ER enzymes involved in collagen I PTMs, thus impairing its post-translational processing and leading to its undermodification. Possible interactions of SPARC with HSP47 and BiP, forming complexes with collagen chaperone functions, are indicated by dashed rectangles. Current research have not shown collagen overmodification caused by OI-like PDI mutations but further research is needed. This figure has been generated using BioRender.

Point mutations substituting one of the glycines in the α1(I) and α2(I) chains represent the most common cause of over-hydroxylation and over-glycosylation of collagen I [35,53,54]. These mutations introduce bulkier amino acids that cannot properly fit within the inner space of the triple helix, prolonging the kinetics of chain folding and increasing their exposure to post-translational modification events [55–57] (Figure 1). Overmodified collagen is partially retained in the cells from OI patients and murine models [55,58–62].

Patients carrying glycine substitutions may present mild, moderate, severe, or lethal OI. Mild OI is typically characterized by near-normal stature, early childhood fractures, blue sclerae, variable hearing loss, and DI [30]. Moderate OI is characterized by short stature with variable skeletal deformities and white sclerae, while severe OI involves progressively worsening bone impairments over time until assistance devices are needed [63]. Affected children may also have a larger than normal head, grayish sclerae, a triangle-shaped face, a deformed chest and spine, and breathing and swallowing problems. Lethal forms present with undertubulation of long bones, prenatal fractures, and dark to blue-gray sclerae; lethality is generally perinatal [64].

It has been consistently reported that glycine substitutions in the α1(I) chain result in a more severe phenotype than substitutions in α2(I) [30,31,65]. Collagen I stoichiometry, that is characterized by two α1 and one α2 chains, provides a partial explanation for this, indeed, mutations in the *COL1A1* result in approximately 75% of collagen molecules being affected, whereas mutations in *COL1A2* affect only 50% of the molecules [30,31,65].

In both chains, the position of the substituted glycine may be relevant with mutations towards the carboxyl-terminal end being more severe than defects in other positions, but several exceptions to this rule weaken its significance for phenotype prediction [56,66,67]. More relevant to modulate clinical severity seems to be the type of glycine substituting amino acid. Residues with charged and bulkier side chains are more destructive for collagen I folding and often result in a more severe outcome. Also, substitution of α1(I) glycine located in specific collagen I helical regions named major ligand binding regions (MLBRs), known to be specific interaction sites of collagen and non-collagenous proteins in the bone ECM, is often associated with more severe patient outcomes [31]. Less understood is the extent to which α2(I) mutations disrupt the stability of the collagen helix and the effect of the position and/or type of substitution [65]. Although mutations in the α2(I) chain were previously thought to cause lethality through a regional model involving eight specific clusters, recent data show that these regions now include a significant number of nonlethal OI cases; however, within seven of them remain discrete stretches associated with disproportionately severe phenotypes, likely due to mutations in residues critical for proteoglycan binding [31].

Splice site variants resulting in exon skipping represent the second most common cause of dominant OI and are also associated with overmodified collagen synthesis [68,69]. They can either result in exon skipping or in intronic inclusion or exonic deletion due to activation of cryptic splice sites [65,70]. Interestingly, exon skipping in *COL1A2* is more likely responsible for lethal phenotype [31].

Even if rare, *COL1A1/COL1A2* deletions or duplications of one or two Gly-Xaa-Yaa triplets can cause OI. These defects are responsible for a shifting of the triple helical registry, delaying α chain folding and impairing N-procollagen processing. Patients carrying these mutations generally present severe clinical outcomes even if the phenotype may vary depending on the size and location of the alteration [71,72].

### Mutations in C- and N- propeptides and signal peptide in *COL1A1*/*COL1A2*

Crystallization studies of the collagen I C-propeptide domain revealed its flower-like shape, characterized by a stalk, a base, and three petals [73]. Proα chain recognition and binding start in the base and petal regions that contain cysteine residues involved in intra- and inter-chain disulfide bonds and the chain recognition sequences. In the base, there is a calcium binding site, critical for stabilization of the trimer [73,74].

The most frequent OI causative mutations in the C-propeptide are substitutions followed by insertions/deletions or splicing errors [74,75]. Clinical outcomes vary from mild to lethal. Procollagens with *COL1A1* C-propeptide defects present delayed chain incorporation, slow folding, and collagen I over-modification [75–77]. Consequently, pericellular processing is defective and fibrils present altered diameters and impaired organization [75]. A more severe outcome is expected when substitutions are in the base or petal regions and disrupt inter-/intra- chain disulfide bonds or calcium-binding sites and a milder outcome when are located at the surface in regions with few interactions [30,74].

Interestingly, a mutation that substituted the first cysteine that participates in intra-chain bonds (proα1(I)Cys1299Trp) caused only mild OI. Secreted procollagen was overmodified but had normal thermal stability, indicating that other cysteine residues in the C-propeptide may have a more important role for C-propeptide assembling [71]. Pathogenic variants in proα2(I) were also described, but they are less common and generally associated with mild OI [74].

In the *Aga2* mouse, carrying a dominant frameshift mutation in the *Col1a1* C‐propeptide, a severe to lethal phenotype associated with bone fractures and decreased bone mass was described. Abnormal proα(I) chains accumulated intracellularly in mutant fibroblasts inducing ER stress, unfolded protein response (UPR) activation, and messenger Ribonucleic Acid (mRNA) up-regulation of BiP and HSP47 [78,79].

Abnormal collagen I structure has also been found due to mutations in the proα1(I) signal peptide. There are two reports of a Gly22Arg substitution causing lethal OI characterized by the synthesis of overmodified intracellular collagen and enlargement of ER cisternae [80].

Some mutations in the N-propeptide domain on proα1(I) have also been described as causative of OI and patients can exhibit the full spectrum of phenotypes, ranging from mild to lethal [81–83]. Nevertheless, the impact on collagen PTMs is unknown [82,84].

### Mutations in the 3-hydroxylation complex

Physiologically, the α1(I)Pro986 residue undergoes C3 hydroxylation mediated by a 3-hydroxylation complex localized within the ER, comprising P3H1, CRTAP, and CyPB [85–87]. P3H1 is the enzyme responsible for the 3-hydroxylation, whereas CRTAP has chaperone function and CypB acts as a PPIase. The KDEL motif (Lys-Asp-Glu-Leu) at the C-terminus of P3H1 is essential for the complex retention and retrieval to the ER, thereby ensuring its proper localization and enabling its function [88]. α1(I)3-Hyp986 is crucial for the proper triple helix folding, for collagen I extracellular cross-linking and fibrils organization [87,89,90].

Null and point mutations in the genes encoding for the three components of the complex cause recessive OI mostly characterized by overmodified collagen I (Figure 1). Defects in *CRTAP* lead to OI type VII [85] generally associated with lethal outcome. Patients present with rhizomelia, fractures at birth, white or light blue sclerae, and osteopenia [87]. *Crtap-*null mice reproduce the phenotype with increased perinatal lethality, rhizomelia, severe osteopenia, and decreased osteoid formation. Also, bone volume/tissue volume (BV/TV), trabecular number (Tb.N), and cortical thickness are significantly reduced with an increase in the hydroxylysylpyridinoline/lysylpyridinoline (HP/LP) cross-link ratio [91,92]. In humans, biallelic *CRTAP* mutations resulted in severe to lethal OI, and newborns frequently display life-threatening respiratory distress, showing also the presence of extraskeletal manifestations. *Crtap* ^−/−^ mice presented as well enlargement in the alveolar airway space in lungs and increased TGF-β activity [93,94].

Mutations in *P3H1* are responsible for OI type VIII [95]. Most of them are frameshift or nonsense mutations that cause significant reduction or absence of *P3H1* mRNA [48]. Clinical features resemble the ones described for *CRTAP* defects, and patients present mostly perinatal lethality and are characterized by rhizomelia. Also, unusual dental abnormalities have been described. A knockout mouse model for this gene showed a similar phenotype with decreased trabecular bone mineral density (BMD). These mice showed overmodified collagen I associated with delayed secretion [96].

Although less common, some missense variants in the catalytic site of *P3H1* have also been reported. They caused multiple long bone fractures, but no other features, overall resulting in less severity. Some authors have suggested that this phenotype may result from reduced enzymatic activity rather than a complete lack of hydroxylation at Pro986. Moreover, the KDEL retention signal and complex stability appear to remain intact [97].

In addition to its role in hydroxylating α1(I)Pro986 and stabilizing collagen I structure, animal studies suggest the complex also functions as a collagen I chaperone. Notably, zebrafish that naturally lack Pro986 3-hydroxylation, in the absence of a functional modifying complex, exhibit a severe skeletal phenotype [88]. Similarly, knock-in mice with a Pro986Ala substitution display bone defects, albeit milder than those observed in *P3h1^-/-^* models [87].

Of note, CRTAP and P3H1 are mutually protecting each other, and the absence of one affects the formation of the complex explaining the phenotypic similarities between type VII and VIII OI [87,98].

Mutations in peptidylprolyl isomerase B (*PPIB*), encoding CyPB, cause recessive OI type IX [99]. The wide phenotypic spectrum in these patients resembles type VII and VIII OI with severe bone deformities, including bowed long bones, scoliosis, and joint hypermobility, though generally less severe and without rhizomelia [98]. Collagen I PTMs in OI type IX are controversial. Fibroblasts isolated from three severely affected children with *PPIB* null mutation showed overmodified procollagen. Authors proved the presence of the mutant procollagen accumulation in the ER complexed with PDI and P4H1 [100]. Interestingly, absence of CyPB did not completely prevent the enzymatic function of the complex because some levels of α1(I)3-Hyp986 were found in one patient (30%) and normal levels in another [100]. Unexpectedly, in other OI type IX patients carrying an Arg-to-Met substitution in the start codon, the absence of CyPB did not delay protein folding and did not alter the proline 3 hydroxylation level [101]. *Ppib* knockout mice displayed increased glycosylation associated with delayed collagen I folding, impaired cross-linking, reduced collagen deposition, abnormal fibril morphology, and diminished bone strength [102]. Unlike human samples, *Ppib^−/−^* mice completely lacked α1(I)3-Hyp986 [103].

### Mutations in *SPARC*

Secreted protein acidic and rich in cysteine (SPARC), also known as osteonectin, is a secreted glycoprotein that binds collagen and other proteins in the ECM regulating collagen fibril formation, stabilization, and ECM mineralization [104–106]. Its presence has also been reported *in vitro* in the nucleus and cytoplasm compartments in murine lens epithelial cells [107] and in the human osteoblastic HOBIT cell line [108]. Pathogenic variants in *SPARC*, identified so far in eight patients, included predominantly missense mutations followed by splice site and nonsense mutations [109–112]. These mutations lead to recessive OI type XVII, a very rare form of the disease characterized by severe clinical presentation that includes bone fragility with fractures, delayed motor development, impaired motor skills, and in most cases, absence of DI [109]. Presence of hypermineralized bone tissue is a common finding in these patients due to excessive collagen cross-linking. Evidence regarding the level of post-transcriptional modifications is limited. Biochemical analysis was performed only in two patients, carrying two homozygous missense mutations (Arg166His and Glu263Lys) in the SPARC binding site for collagen I. Both showed reduced or normal SPARC translation, mildly overmodified collagen, and severe bone phenotype [109].

The mechanisms behind these observations are poorly understood. It has been proposed that intracellular SPARC acts in concert with HSP47 to ensure that only correctly folded procollagen molecules exit the ER, which could explain the modified PTMs, but deeper investigations are needed [113]. Of note, a recent mass spectrometry study in colorectal cancer confirmed the interaction between SPARC and BiP [114], a heat shock protein 70 (HSP70) molecular chaperone found in the ER, that facilitates proper folding and oligomerization of newly synthesized proteins and targets misfolded or unassembled proteins for proteasomal degradation [115]. This could be another mechanism implicated in OI type XVII highlighting SPARC's role in driving ER stress-induced apoptosis and UPR activation (Figure 1). SPARC-null mice presented smaller fibrils with osteopenia, decreased bone formation, and bone remodeling [116], but to our knowledge, no specific investigation on collagen I PTMs has been reported to date.

## OI characterized by undermodified collagen I

### Mutations in *TMEM38B*

Transmembrane protein 38, member B (*TMEM38B*) encodes an ER trimeric intracellular cation channel type B (TRIC-B) that allows the flux of potassium as counter-ion for the Ca^2+^ efflux from the ER to the cytosol through the inositol 1,4,5-triphosphate receptor (IP3R). Thus, TRIC-B is involved in maintaining calcium homeostasis [117–119].

Null mutations but also splice sites and substitutions in *TMEM38B* sequence are causative for OI type XIV [120,121]. This form is characterized by an extremely wide clinical outcome. OI type XIV patient phenotypes range from mild to severe, and the most common features are bone fragility, fractures, bowed limbs, and osteopenia. In contrast to most of the other forms of OI, bone in OI type XIV is not hypermineralized, and bone turnover is low rather than high, as seen in dominant OI [122]. Cardiovascular manifestations appear in this type of OI. Loss of function mutations caused atrial and ventricular septal defects, heart failure, tricuspid regurgitation, asymmetric septal hypertrophy of the left ventricle, and mild aortic root dilation in patients [123]. Similar findings in mice showed that double knockout mutations in TRIC-A and TRIC-B cause embryonic lethality due to cardiac arrest, and *Tric-a^−/−^;Tric-b^+/−^* are susceptible to stress-induced heart failure [124]. Ca²^+^ ions are essential for the proper folding and assembly of collagen I C-propeptide domain by facilitating inter- and intra-chain disulfide bond formation [73]. TRIC-B absence impaired the release of ER luminal Ca^2+^, which disrupts the activity of ER-resident calcium-binding proteins such as calreticulin and calnexin, necessary for collagen maturation, and of P4H1 and LHs, which require a calcium-rich environment to catalyze hydroxylation reactions of procollagen chains. Consequently, patient fibroblasts and osteoblasts presented undermodified collagen I (Figure 1) likely due to a reduction in helical lysine hydroxylations, despite delayed procollagen assembly and increased telopeptide hydroxylation [125]. Biochemical analysis in a bone-specific conditional *Tmem38b* knockout mouse confirmed the reduced collagen I PTMs [122].

In OI type XIV, most of the misfolded collagen is retained intracellularly, causing ER stress and increased BiP, and the amount of secreted collagen is significantly reduced, resulting in matrix insufficiency (50-70%) [125]. In osteoblasts, ER stress due to Ca^2+^ dysregulation has been related to elongated mitochondrial and increased generation of reactive oxygen species (ROS), revealing a possible implication of mitochondria in this OI form. *TMEM38B* mutations also impair gap and tight adhesions of osteoblasts, cell proliferation, and cell cycle [126,127].

Lack of Tric*-b* specifically in murine osteoblasts revealed that the compromised Ca^2+^ homeostasis negatively impacted Ca^2+^ calmodulin kinase II (CaMKII) activity compromising the suppressor of mother against decapentaplegic (SMAD) signaling pathway responsible for impaired osteoblast differentiation [122]. Conditional knockout mice also showed altered cytoskeleton assembly with β-catenin accumulation at osteoblast adhesion sites [128].

## OI characterized by unaltered collagen I

### Null mutations in *COL1A1*/ *COL1A2*

**Figure 2:**
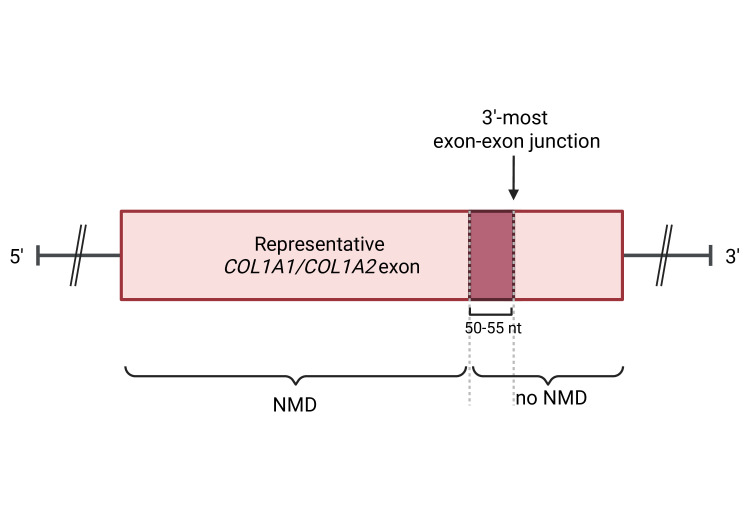
Impact of PTC position on NMD activation in collagen I genes. Representative *COL1A1/COL1A2* exon showing the 50–55 nucleotides NMD rule. Premature termination codons (PTCs) upstream the 50–55 nucleotides of the most extreme exon-exon junction activates nonsense-mediated decay (NMD) impeding the translation of the mutant chain and leading to haploinsufficiency. In contrast, when PTC causing mutations are within or downstream this region, NMD is not activated allowing the translation of defective procollagen chains. This figure has been generated using BioRender.

Genetically, null *COL1A1* mutations can arise from various variants, including nonsense mutations, frameshift mutations that disrupt the reading frame, and splice site mutations that introduce premature termination codons (PTCs) [66]. These mutations often activate nonsense-mediated decay (NMD), a cellular mechanism that degrades defective mRNA, thereby preventing the production of incomplete or aberrant collagen chains [129,130]. The activation of NMD depends on the position of the PTC. A general rule, which has also been confirmed for collagen genes [74,131], is that only PTCs located more than 50–55 nucleotides upstream of the final exon–exon junction can trigger NMD. In contrast, PTCs located within the last 50–55 nucleotides before the final exon–exon junction, or within the last exon of the gene, often escape NMD [131–133] (Figure 2). Activation of NMD in *COL1A1* leads to haploinsufficiency, a condition in which one functional copy of the gene is insufficient to produce adequate collagen, resulting in a 50% reduction in the overall amount of normal collagen I [134]. This quantitative deficiency is classified as OI type I and underlies the milder clinical manifestations. Patients typically present with mild bone fragility, normal or slightly reduced stature, frequent fractures, particularly during childhood, although the incidence of fractures often decreases after puberty. A distinctive feature is the presence of blue sclerae, caused by the thinning of connective tissue in the eye, which allows pigmented tissue and blood vessels to show through [30,129]. On the other hand, PTCs that do not activate NMD end up with the production of stables and generally overmodified procollagen I chains that cause a more severe phenotype [74]. Heterozygous nonsense mutations in *COL1A2*, which trigger NMD and consequently result in the functional loss of one allele, generally do not manifest a phenotype due to compensation by the remaining allele. In contrast, homozygous mutations subject to NMD in *COL1A2* lead to a distinct cardiovascular form of Ehlers-Danlos syndrome (EDS type VIIB), without bone involvement [135].

### Mutations in *COL1A1*/*COL1A2* encoding the C- and N-telopeptide cleavage sites and in *BMP1*

Dominant mutations in the C-telopeptide cleavage site of proα chains result in the synthesis of properly glycosylated procollagen molecules that exhibit impaired extracellular processing. The absence of a suitable substrate for the BMP1 C-proteinase leads to the synthesis of collagen molecules retaining the C-propeptide (pC-collagen), which are incorporated in the ECM assembly in abnormal fibrils [74,75]. These defects are responsible for milder OI forms characterized by high bone density [75]. Cellular studies showed that procollagen with normal post-translational modified triple helix still exhibits delayed secretion into the ECM [136].

**Figure 3:**
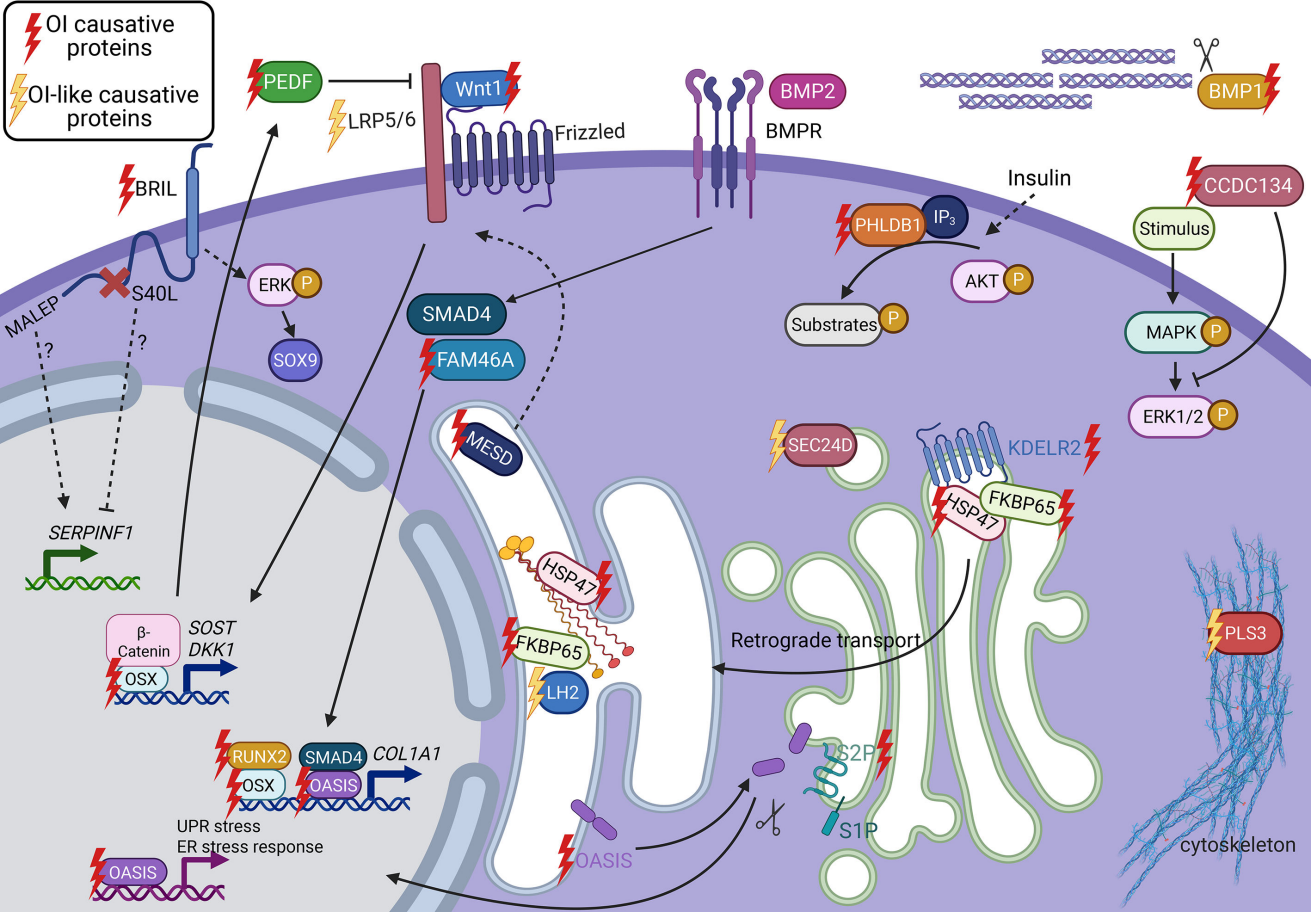
Proteins involved in OI without altering collagen I structure. During collagen synthesis in the ER, HSP47 and FKBP65 interact with procollagen molecules to ensure proper folding and cross-linking. FKBP65 also complexes with LH2 and regulates its hydroxylation activity. The KDEL receptor 2 (KDELR2) recognizes HSP47 and FKBP65 and allows their retro-translocation from the Golgi to the ER. OASIS is an ER transmembrane protein that relocates to the Golgi upon stress conditions, where its transcriptional factor domain is released by S2P cleavage and activates the transcription of UPR and ER stress responses genes in the nucleus. OASIS is also important for the expression of SEC24D, a COPII vesicle component involved in collagen secretion. OSTERIX (OSX) is another transcription factor crucial for early osteoblasts differentiation. It also modulates the WNT signaling pathway by inducing the expression of WNT inhibitors, for example *SOST* and *DKK1*. The WNT pathway is activated by the binding of WNT1 ligand to the LRP5/6 receptor. Within this pathway, MESD is an ER chaperone for the WNT receptors LRP5/6. BRIL is a transmembrane protein that, when mutated, activates the ERK/MAPK pathway ultimately leading to accumulation of SOX9, the master regulator of chondrogenesis. BRIL mutations have also been linked to inhibition of *SERPINF1* encoding PEDF, that binds collagen I in the ECM. BMP1 cleaves the C-propeptide of collagen I. CCDC134 is involved in the regulation of the MAPK/ERK signaling. PHLDB1 facilitates the insulin-dependent activation of protein kinase B/Akt. FAM46A post-transcriptionally regulates collagen mRNA. PLS3 is a cytoskeletal protein. This figure has been generated using BioRender.

Retention of C-propeptide in collagen I molecules can also be due to recessive mutations in the BMP1 enzyme (Figure 3). In this case, the primary sequence of collagen I is intact, but propeptide cleavage defects lead to ECM accumulation of pC-collagen, and this causes OI type XIII. Even if patients can present a range of clinical outcomes, the phenotype is generally more severe than what is reported for mutations in the cleavage sites, likely since lack of BMP1 affects not only procollagen I processing, but also other ECM proteins such as lysyl oxidase (LOX), type II and III procollagens, and other secreted factors [137]. OI type XIII patients show high bone mineral density and disrupted fibril assembly and mineralization, as patients with defective C-propeptide processing [138–140].

Most mutations in the N-propeptide cleavage site or in the first residues of the helical region of proα1(I) are associated with a form of OI/EDS [141,142]. These defects primarily delay N-propeptide cleavage. The incorporation of mutant pN-collagen into the matrix leads to reduced stability and fibril diameters [142]. Patients diagnosed with OI/EDS present mild OI, with bright blue sclerae, fractures, and hyperextensibility of large and small joints. The phenotypic severity is correlated with the substituted residue and its distance from the cleavage site [141].

### Mutations in proteins involved in collagen folding, cross-linking, and trafficking: HSP47, FKBP65, LH2, KDELR2, PH4B, OASIS, SEC24D, and S2P

Mutations in proteins/enzymes directly involved in the collagen folding, cross-linking, and trafficking from the ER to the extracellular space cause the synthesis of normally glycosylated collagen I molecules, but with reduced secretion efficiency. During collagen processing in the ER, the two chaperones HSP47 and 65 kDa FK506 binding protein (FKBP65) interact with procollagen molecules to ensure proper folding and cross-linking, respectively (Figure 3) [16]. HSP47, encoded by serpin family H member 1 (*SERPINH1*), is a collagen-specific molecular chaperone that is co-expressed with collagen [15]. It binds to procollagen in the ER in a pH-dependent manner [143] and facilitates folding of procollagen chains. HSP47 remains bound to procollagen until the protein translocates to the *cis*-Golgi, where the lower pH favors its dissociation [144]. Mutations in HSP47 result in the extremely rare recessive OI type X [145–147]. Patients show a severe to lethal phenotype, with severe skeletal defects, thin ribs with multiple fractures, deformed long bones, macrocephaly, and blue sclerae. Most patients suffer from respiratory issues that cause early death [145–147]. Still, patients and knockout *Hsp47* mice produce normal collagen I, although characterized by delayed secretion with consequent accumulation in the ER and increased susceptibility to protease cleavage [145,148]. Recently, a child with OI type X was found with overmodified collagen I, despite normal folding and secretion, alongside the up-regulation of several other procollagen chaperones, including FKBP65 [147]. FKBP65, encoded by FK506 binding protein 10 (*FKBP10*) gene, acts as chaperone for the formation of the collagen triple helix, and it has a peptidylprolyl *cis-trans* isomerase activity directly involved in collagen cross-linking and strictly related to the function of LH2 [16,149]. FKBP65-LH2 complex regulates LH2 hydroxylation activity [149]. Indeed, mutations in *PLOD2*, encoding LH2, cause an OI-like phenotype overlapping with OI type XI and classified as Bruck type 2 syndrome also referred to as OI with congenital joint contractures. In this OI and OI-like forms, collagen type I is characterized by underhydroxylation of the lysine residues in telopeptides [150,151]. Consequently, the normal folded collagen I shows reduced cross-linking and decreased deposition in the matrix [152]. Mutations in *FKBP10* have been linked to either recessive OI type XI, Bruck syndrome, or Kuskuskwim syndrome [153–157]. Interestingly, mutations in *SERPINH1* not only decrease the levels of HSP47, but also reduce the expression of FKBP65, although the opposite does not occur, supporting a co-operative action of HSP47 and FKBP65 in procollagen trafficking from the ER to the Golgi [158].

Being ER resident chaperones, HSP47 and FKBP65 are retro-translocated from the Golgi to the ER thanks to the specific retrograde transport that requires coat protein complex I (COPI)-coated vesicles and occurs via the recognition by KDEL receptors of the C-terminal KDEL motif in a pH-dependent manner [159–162]. Both HSP47 and FKBP65 have a KDEL-like domain recognized by the KDEL receptor 2 (KDELR2) (Figure 3) [63,163]. Mutations in KDELR2 result in a decrease of HSP47 and FKBP65, a decreased collagen secretion and ultimately the lack of proper collagen fiber assembly, given that HSP47 was found associated with collagen molecules in the ECM [163]. The absence of KDELR2 cannot be compensated by the other KDEL receptors and leads to recessive OI type XXI, characterized by reduced intracellular levels of HSP47, which in turn causes a decrease in intracellular FKBP65, thereby overlapping with OI types X and XI [163].

P4HB, which encodes the β subunit of prolyl 4-hydroxylase (also known as PDI), acts as both a catalyst for proline hydroxylation within the Xaa-Pro-Gly repeats of the procollagen helical domain and as a molecular chaperone, assisting in proper collagen folding and stabilization in the ER. Mutations in this gene cause a distinct OI-like form known as Cole-Carpenter syndrome, characterized by frequent fractures, craniosynostosis, ocular proptosis, hydrocephalus, and distinctive facial dysmorphisms [164,165]. Heterozygous missense mutation in *P4HB* exon 9 affected the disulfide isomerase activity of PDI *in vitro* causing increased ER stress due to collagen accumulation, but normal PTM pattern and secretion rate of collagen I α chains [165]. Nevertheless, heterogeneity is also reported for this gene because another novel heterozygous missense mutation, namely c.692A > C in exon 5, led to mild OI in two patients [166]. Further research is needed to clarify the status of collagen PTMs in these patients.

The ER, and in particular, the activation of ER stress upon misfolded protein accumulation, has a crucial role in the pathophysiology of OI. Among the ER stress sensors, the old astrocyte specifically induced substance (OASIS), encoded by the cAMP responsive element binding protein 3-like 1 (*CREB3L1*), is a transmembrane ER-resident basic leucine zipper (bZIP) transcriptional factor that belongs to the AMP response element-binding protein/activating transcription factor (CREB/ATF) family [167]. Under stress conditions, OASIS relocates to the Golgi membrane where it undergoes proteolytic cleavage that releases its N-terminal cytoplasmic domain [168] that shuttles to the nucleus to activate the transcription of genes involved in the ER stress response or the UPR pathway, and it also binds to *COL1A1* promoter via SMAD4 to increase collagen I expression (Figure 3) [169]. Recessive mutations in *CREB3L1* result in OI type XVI that shows phenotypes with variable severity [170–173]. Mutant OASIS cannot bind the UPR element-like sequence in the *COL1A1* promoter [171]. Thus, absence of OASIS causes reduced collagen I in osteoblasts and bone, generating osteopenia and fractures in patients and mice [168]. Moreover, OASIS is important for the expression of the COPII component Sec24 homolog D (SEC24D), involved in collagen trafficking and secretion [171]. *SEC24D* mutations lead to Cole Carpenter syndrome type 2 associated with procollagen retention in the ER, showing an OI overlapping phenotype [174]. Recent findings have linked mutations in *SEC24D* to defects in osteogenic differentiation, possibly due to inactivation of the activating transcription factor 6 / transforming growth factor beta/ runt-related transcription factor 2 (ATF6/TGF-β/Runx2) regulatory loop [175].

Of note, OASIS cleavage occurs in the Golgi by the Regulated Intramembrane Proteolysis (RIP) accomplished by the site 2 metalloprotease (S2P), that catalyzes the second cleavage reaction of OASIS, following S1P cleavage (Figure 3) [169,176]. Recessive mutations in the *MBTPS2*, encoding S2P, lead to X-linked moderate OI type XVIII and other syndromes based on the location of the mutation [177]. In OI, *MBTPS2* mutations result in a reduced collagen expression and impaired collagen cross-linking, given that the Lys87 hydroxylation in both α(I) chains is reduced by 50% [177].

### Mutations in proteins critical for osteoblast differentiation and function: OSTERIX, WNT1, LRP5, MESD

Normal post-translationally modified collagen characterizes also a group of OI caused by defects in genes encoding proteins necessary for osteoblast differentiation and activity.

Among them, OSTERIX encoded by *SP7* is expressed in immature osteo-chondro-progenitors and osteoblasts upon Bone Morphogenetic Protein 2 (BMP2) stimulation/ER stress [178,179]. *SP7* deletion in mice results in absent bone development due to lack of osteoblast differentiation, causing perinatal death [178]. At the cellular level, OSTERIX interacts with RUNX2 to induce *COL1A1* expression (Figure 3) [180]. It also modulates the wingless-related integration site (WNT) signaling pathway by inducing the expression of WNT inhibitors, i.e. sclerostin (*SOST*) encoding SCLEROSTIN and dickkopf-related protein 1 (*DKK1*) encoding DICKKOPF [181–183]. Homozygous or heterozygous mutations in *SP7* cause the extremely rare OI type XII, either with recessive [111,184–186] or dominant inheritance [187] and mostly characterized by moderate bone fragility and deformity, delayed eruption of teeth, normal sclerae, and variable DI [30].

In the context of osteoblast differentiation and function, the WNT/β-catenin is a major signaling pathway regulating bone development [30]. The WNT pathway is activated by the binding of WNT ligands to the frizzled/low density lipoprotein receptor-related protein 5 or 6 (LRP5/6) complex at the cell surface (Figure 3). These receptors transduce a signal that prevents phosphorylation and subsequent intracellular degradation of β-catenin. Thus, upon WNT activation, β-catenin accumulates in the nucleus, where it forms a transcriptional complex with T-cell factor (TCF)/lymphoid enhancer-binding factor (LEF) and induces expression of target genes including *RUNX2* and *SP7* [188–190]. Mutations in the WNT family member 1 (WNT1), specifically expressed by osteocytes [191,192], cause recessive OI type XV. Homozygous mutations in WNT1 are responsible for a moderate to severe phenotype characterized by short stature, multiple vertebral compression fractures, kyphoscoliosis, and long bone fractures [191,193–202]. A peculiarity of this OI subtype is that it combines central nervous system anomalies with the bone phenotype [200,203]. OI type XV patients frequently present with brain malformations, with prominent brainstem and cerebellar hypoplasia alongside severe intellectual and motor deficits. It has been proposed that most brain anomalies in WNT1-associated OI have vascular origins related to the role for WNT1 in central nervous system angiogenesis [200].

Interestingly, heterozygous dominant mutations in WNT1 cause early-onset osteoporosis [191,194,204,205]; likewise, mutations in *LRP5* result in both dominant and recessive forms of osteoporosis [206]. Either mutations in *WNT1* or *LRP5* compromise the binding of WNT1 protein to the Frizzled/LRP5 or 6 receptors, thus affecting the WNT pathway activation and ultimately impairing osteoblast function and bone homeostasis [191,194,205].

Recently, mutations in the mesoderm development candidate 2 (MESD) encoding an ER chaperone for the WNT receptors LRP5/6 have been linked to the recessive OI type XX (Figure 3) [207–210]. Mutant MESD protein cannot be retained in the ER, leading to the mislocalization of LRPs, which aggregate in the cytoplasm and cause the loss of signaling in the canonical WNT pathway [207,210,211]. Furthermore, MESD has been shown to be also a direct chaperone of collagen I [211]. Thus, cells with mutant MESD are characterized by intracellular collagen I aggregates and significant collagen retention, resulting in increased autophagy and overall increased cellular stress [211]. Patients suffer from a progressive deforming skeletal dysplasia with recurrent fractures, short stature, rhizomelia, progressive scoliosis, and kyphosis with vertebral compressions [207,209,212]. By the second decade of life, patients also develop dental abnormalities [207,209]. Differently from other OI forms, MESD mutations cause reduced and inhomogeneous bone matrix mineralization [208]. The more severe phenotypes are linked to a complete protein loss of function, thus suggesting a role of MESD in early skeletal development [210,213].

### Mutations in proteins involved in bone matrix mineralization: BRIL, PEDF, PLS3

The bone-restricted interferon-induced transmembrane protein-like protein (BRIL), highly expressed in osteoblasts, was identified as a player in the mineralization process [214]. Interestingly, the expression of *IFITM5*, encoding BRIL, increases during osteoblast differentiation, peaking with matrix production and mineralization, although its specific function remains to be fully understood [214,215]. A recurrent gain of function mutation in the 5’-UTR of *IFITM5* (c.-14C > T) causes the only dominantly inherited OI besides the classical forms, namely OI type V [216,217]. The mutation generates a new upstream start codon resulting in the addition of five amino acids (MALEP) to the N-terminus of BRIL (named MALEP-BRIL) (Figure 3) [216,217]. The elongated MALEP-BRIL is stable, localizes at the cell membrane, and shows no differences in synthesis levels compared to BRIL [218]. In addition, osteoblasts from patients cultured *in vitro* demonstrated increased osteoblast maturation markers, such as osteopontin (*SPP1*), alkaline phosphatase (*ALPL*), and integrin-binding sialoprotein (*IBSP*) as well as decreased expression of *COL1A1* [215]. These data, together with the increased levels of serum alkaline phosphatase in patients, support the higher mineralization that characterizes this OI form [219]. Patients also have increased osteocyte number and size and irregular bone lamellae [219]. Likewise, transgenic mice overexpressing *Ifitm5* in osteoblasts are perinatal lethal with skeletal defects, fractures, and impaired *in vitro* osteoblast mineralization [220]. Furthermore, recent findings in two murine models demonstrate that *Ifitm5* mutations likely cause the downstream activation of extracellular signal-regulated kinase/mitogen-activated protein kinase (ERK/MAPK) and elevated SRY-box transcription factor 9 (SOX9) protein, the master regulator of cartilage development. This results in chondrogenesis defects and impaired mineralization, characteristic of OI [221].

A different heterozygous mutation in *IFITM5* (c.119C > T) results in the so-called atypical OI type V. This mutation affects two palmitoylation sites of BRIL (S50 and S51 in humans, S52 and S53 in mice) that causes poor palmitoylation of the protein that is retained in the Golgi [218]. Interestingly, patients present a clinical phenotype overlapping with OI type VI, caused by mutations in serine protease inhibitor, family F, member 1 (*SERPINF1*) and encoding the secreted glycoprotein pigment epithelium-derived factor (PEDF) (Figure 3) [222,223]. PEDF is an anti-angiogenic cytokine that exerts its function upon binding to collagen I in the ECM [224–226]. PEDF also favors osteogenesis and inhibits adipogenesis, thus enhancing the differentiation of mesenchymal stem cells into osteoblasts [227,228]. The mechanisms leading to OI type VI remain to be elucidated, but patients suffer from frequent fractures after one year of age, persistently elevated serum alkaline phosphatase in childhood, and absence of serum PEDF. The bone exhibits fish-scale-like lamellae and excessive accumulation of osteoid. Although the bone is hypermineralized, it is surrounded by areas of low mineral content and an increased number of osteocytes [229,230]. Similarly, patients with atypical OI type V have elevated serum alkaline phosphatase in childhood, under mineralized osteoid and fish-scale lamellae in bone, although normal PEDF in serum [231]. The phenotypic overlap between OI type VI and atypical OI type V suggests a cross-talk of the pathways involving BRIL and PEDF, thus converging in a biochemical and molecular cascade of events that ultimately lead to OI [30].

Another protein, plastin-3 (PLS3), encoded by *PLS3*, has a role in bone formation, mineralization, and resorption. PLS3 is an ubiquitous cytoskeletal protein involved in the formation of F-actin bundles and known to be expressed in osteoblasts, osteocytes, and osteoclasts [232–235]. PLS3 likely participates in the mechano-sensing function of osteocytes [236] and is also involved in the regulation of the nuclear factor kappa-light-chain-enhancer of activated B cells (NF-κB) pathway, thereby suppressing osteoclast function. Mutations in PLS3 result in reduced trabecular thickness despite normal expression and posttranslational modification of collagen I, as well as preserved overall bone structure. Consequently, the osteoporosis and OI-like phenotype associated with PLS3 mutations may primarily arise from increased osteoclast activity [236,237].

### Mutations in protein involved in post-transcriptional regulation of collagen mRNA: FAM46A

The terminal nucleotidyltransferase 5A (*TENT5A*) gene encodes the family with sequence similarity 46, member A (FAM46A), a member of the superfamily of nucleotidyltransferase fold proteins that catalyze the non-canonical polyadenylation of transcripts, which are conserved in all known animal genomes [238]. FAM46A is highly expressed in mouse embryonic skeleton and human osteoblasts (Figure 3) [239,240], and murine studies confirmed that it is responsible for the polyadenylation of *Col1a1* and *Col1a2* transcripts [241]. *Fam46a* null mice have a distinctive skeletal dysplasia, with small size, long bone abnormalities, bent and twisted limbs, compressed rib cage, bone fragility, and reduced trabecular and cortical bone [239]. The collagen in the mouse bone tissue is characterized by thin and disorganized fibrils, and osteoblasts exhibit a deficiency in collagen secretion [241]. Patients with recessive mutations in *TENT5A* also show congenital bowing of limbs, early fractures, blue sclerae, hyperlaxity, and motor delay, a condition that was classified as OI type XIX [240]. The severe phenotype could be related to a possible broader role of FAM46A. In fact, mouse knockout osteoblasts have significantly shortened poly-A tails in several mRNAs, the most relevant include *Col1a1, Col1a2, SerpinF1,* and *Sparc* [241].

However, FAM46A likely has other functions that remain to be fully understood. In *Xenopus* development, Fam46a physically interacts with SMAD1/SMAD4 proteins to induce transcription of bone morphogenetic protein target genes in the TGF-β pathway [242,243].

### Mutations in proteins participating in signaling pathways: CCDC134 and PHLDB1

The MAPK/ERK pathway promotes early commitment and differentiation of skeletal progenitors to the osteoblast lineage and skeletal mineralization [244]. Upon activation by an extracellular ligand, an intracellular cascade leads to the activation of MAPK that phosphorylates ERK1 and 2. Phosphorylated ERK1/2, in turn, activates by phosphorylation RUNX2 and induces the transcription of target genes such as bone gamma-carboxyglutamate protein 2 (*Bglap2*), integrin-binding sialoprotein (*Ibsp*), and alkaline phosphatase, liver/bone/kidney (*Alpl*) [245]. In addition, ERK1/2 and MAPK can function indirectly by activating secondary kinases that stimulate osteoblast gene expression or, alternatively, inhibit the activity of factors to directly or indirectly suppress osteoblast activity [246].

The coiled-coil domain-containing protein 134 (CCDC134) is a widely expressed secretory protein involved in the regulatory mechanism of the intracellular MAPK/ERK signaling pathway (Figure 3). CCDC134 inhibits ERK1/2 phosphorylation, thus modulating the MAPK/ERK pathway. At the molecular level, defects in CCDC134 result *in vitro* in increased ERK phosphorylation, decreased expression of *COL1A1* and osteopontin, and reduced mineralization [247]. Phenotypically, homozygous mutations in *CCDC134* cause a severe recessive skeletal fragility syndrome that has been classified as OI type XXII [247–249]. Patients present pre- and post-natal short stature, multiple fractures and bowing of long bones, low mineral density, and wormian bones. Patient bone histomorphometry analysis revealed decreased trabecular bone associated with atypical increased cortices, with normal mineral apposition rate and bone formation rate, characteristics of defects in the MAPK/ERK pathway [247]. The mechanism by which defects in *CCDC134* lead to OI may be multifaceted and potentially overlap with other signaling pathways, which remain to be elucidated [30].

Recently, mutations in the pleckstrin homology-like domain family B member 1 (PHLDB1) have been identified in patients affected by a mild OI form, named as OI type XXIII [250]. PHLDB1, encoded by *PHLDB1*, has a role in facilitating the insulin-dependent activation of protein kinase B, also known as Akt (Figure 3) [251,252]. The C-terminal domain of PHLDB1 has affinity for phosphatidylinositol 3,4,5-triphosphate and is thought to facilitate its plasma membrane recruitment for the activation of the AKT kinase [253]. AKT phosphorylates several substrates to regulate proliferation, growth, survival, and metabolism in many cell types [254]. Patients lacking PHLDB1 have recurrent fractures, short stature, bowing of long bones, osteopenia, and *Phldb1* knock-out mice display reduced bone content and mineralization defects [250,255–258]. Although the biological function of PHLDB1 in bone remains to be fully understood, an *in silico* association study suggests that PHLDB1 is linked to LEPREL2, a collagen prolyl hydroxylase involved in collagen synthesis, folding, and assembly [5,250]. Thus, lack of PHLDB1 might indirectly affect collagen biosynthesis. Moreover, PHLDB1 is localized at cell adhesion sites where laminin-5 is present, suggesting a possible role of PHLDB1 in osteogenic differentiation [250,259–261].

## Impact of collagen alterations on cellular and extracellular environments

Alterations in both the structure and abundance of collagen I in OI lead to substantial dysregulation of cellular processes and ECM formation in bone.

### Effects of altered collagen structure or amount in cellular environment

**Figure 4:**
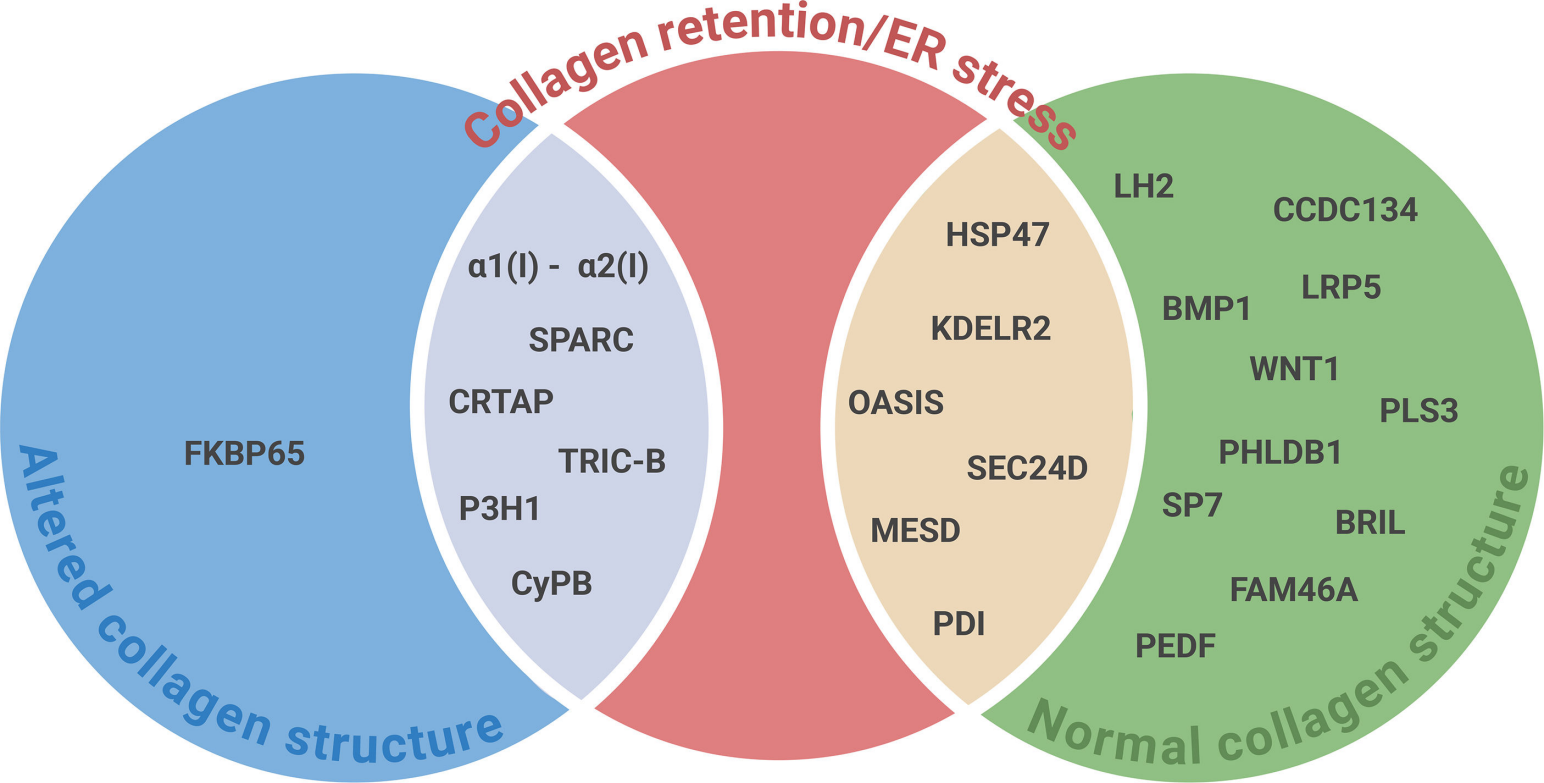
Collagen retention and endoplasmic reticulum stress are common features in OI in the presence of normal and altered collagen structure. This figure has been generated using BioRender.

At cellular level, despite the genetic heterogeneity, accumulating evidence reveals a convergent cellular pathology centered on ER stress and disrupted proteostasis which constitutes a unifying molecular mechanism in the OI pathophysiology, whether associated with aberrant collagen structure or not (Figure 4).

In 14 OI forms, mutations affecting collagen I chains (α1/2), collagen I associated chaperones (HSP47, SPARC, FKBP10), enzyme complexes involved in its post-translational modification (P3H1 complex, P4HB), proteins involved in its intracellular transfer from ER to Golgi (KDLR2, SEC24D), transcription factor regulating its expression (OASIS) or an ER channel involved in modulating calcium flux (TRIC-B) result in intracellular collagen retention. The ER accumulation of collagen I triggers a robust cellular stress response, activating canonical and non-canonical UPR pathways aimed at restoring proteostasis [262,263]. In OI, ER stress and the UPR are variably activated depending on the type of mutation. Variants that impair collagen triple helix assembly often lead to retention of misfolded procollagen chains and up-regulation of the ER chaperone BiP and activation of ER-associated degradation (ERAD), without consistently activating the transmembrane UPR sensors [67]. In contrast, mutations that cause the production of structurally abnormal collagen, including both glycine substitutions and defects in post-translational modifying enzymes (e.g. CRTAP, P3H1, or PPIB), activate the protein kinase R (PKR)-like endoplasmic reticulum kinase (PERK)-ATF4 and inositol-requiring enzyme 1- X-box binding protein 1 (IRE1-XBP1) branches of the UPR [67]. These pathways contribute to reducing the protein load, enhancing folding capacity, and in severe cases, promoting apoptosis [264].

Acting downstream or in parallel to UPR, the Integrated Stress Response (ISR) modulates translation rates of secretory proteins to alleviate ER burden [265]. Interestingly, it has been shown in murine OI osteoblasts that ISR may also be activated and its signaling is regulated via mitochondrial paralogs of UPR components, HSP70 and ATF5, reflecting cross-talk between ER and mitochondrial stress pathways [266]. Mitochondrial dysfunction, including altered morphology, impaired bioenergetics, and defective mitophagy, is increasingly recognized in both dominant and recessive OI models and may exacerbate cellular stress [126,266–268]. In particular, muscle from OI murine models showed mitochondria with evident reduction of respiration rates, biogenesis markers, mitophagy, and electron transport chain components [267]. Interestingly, in a recent study on OI type XIV, osteoblasts showed an aberrant mitochondria morphology, with issues in mitochondrial fission/fusion that resulted in organelle malfunction [126,269,270]. Thus, ER stress, UPR and ISR pathways, and mitochondrial dysfunction can contribute to or mitigate the OI phenotype via a delicate balance, still to be completely elucidated and deserving future investigation [30]. Targeting mitochondrial function to restore proper dynamics and bioenergetics represents a promising therapeutic avenue that could alleviate cellular stress and improve bone quality in OI.

A particular mention should be made of the OI form caused by mutations in MBTPS2, which disrupt the process of RIP. This disruption specifically impairs the activation of transcription factors such as OASIS and UPR-related gene expression. The failure of RIP signaling in this context leads to attenuated UPR activation and a distinct imbalance in cellular homeostasis.

### Effects of altered collagen structure or amount in the extracellular environment

At the extracellular level, OI is characterized by an insufficient amount of bone with abnormal structural organization and impaired properties [34,63,78,163,271,272]. The reduced collagen content in the ECM may consist of collagen that is either structurally normal, but present in decreased amounts, or collagen exhibiting primary structural defects or altered PTMs [273]. One major consequence of the altered collagen structure is its aberrant 3D assembly that negatively affects on fibril formation in the ECM [274]. Increased or decreased levels of Hyl and glycosylated Hyl impair collagen cross-linking, thereby impairing spontaneous fibril formation and compromising the stability of the collagen matrix [275,276]. Altered collagen cross-links weaken bone strength, since they contribute to decreased bone toughness resulting in diminished resistance to microcrack propagation, as demonstrated in the *oim* mouse, one of the most studied OI mouse models that produce α1(I) homotrimers [277].

In OI, the altered collagen fibrils have a smaller diameter and the individual collagen molecules within the fibril are more widely spaced, likely due to the steric hindrance caused by over-modification of collagen I [278,279]. Li et al. [280] found that collagen fibers in *oim/oim* mice were thinner, loosely packed, and had significantly smaller D-spacing, affecting stiffness distribution and intra-fibrillar mineralization [280]. Scanning transmission electron microscopy further showed regions of both organized and disorganized fibrils in *oim* tendons, alongside differences in mineral composition compared to wild-type [281]. In *Brtl* mice, carrying a α1(I)Gly349Cys substitution, a greater variation and altered distribution of collagen D-spacing was observed [282].

Atomic Force Microscopy (AFM) studies on OI models reveal that mutations in collagen I cause nanoscale structural defects in the ECM, including disrupted D-banding periodicity, irregular fibril morphology, and altered mechanical properties like reduced stiffness. These changes impair collagen fibril assembly and weaken bone matrix integrity [283].

Recent advances in cryogenic electron microscopy (cryo-EM) and cryogenic transmission electron microscopy (cryo-TEM) have significantly deepened our understanding of collagen ultrastructure during bone mineralization. High-resolution imaging has revealed that mineralized collagen fibrils undergo axial contraction specifically in the gap regions, coupled with lateral expansion and increased packing density. These structural adaptations facilitate the infiltration and organization of hydroxyapatite crystals without disrupting the characteristic collagen banding pattern. Importantly, these studies have shown that maintaining the delicate ultrastructural arrangement between the organic collagen matrix and the mineral phase is crucial for preserving bone mechanical integrity. Disruptions caused by abnormal PTMs or collagen I mutations can impair this synergy, leading to compromised bone strength and increased fragility [284,285].

Abnormal collagen also affects the interaction with integrins, the major cell surface receptors for collagen I, whose function is to ‘integrate’ the outside with the intracellular environment via biochemical and mechanical signals [286,287]. Integrin-matrix interactions have a strong impact on cell functions, development, homeostasis, and have a role in pathophysiology of disorders, including OI. In particular, integrin binding sites present in collagen have been shown to be important in collagen-induced endothelial cell activation [288], osteoblast differentiation [289], and angiogenesis [290]. Many OI-causing mutations resulting in structurally abnormal collagen I lead to local destabilization of the triple helix and likely impairment of integrin-binding sites [65,291–293]. Compromised integrin-mediated cell-collagen interactions lead to cellular dysfunctions via disruption of the cytoskeleton and of local adhesion in both dominant and recessive OI forms [294,295].

Hypermineralization associated with reduced bone mass is a hallmark for most OI types, both dominant and recessive, with the exceptions of OI type VI with accumulation of osteoid, type XIV and XV with mostly normal mineralization, and type XX in which patients show a reduced bone mineralization [123,208,229–231,296]. The expansion of the space between aberrant collagen molecules may allow for the accommodation of more mineral crystals, which could be a contributing factor to the abnormally high bone matrix mineralization observed [279]. This condition enormously contributes to bone brittleness and fragility in OI. Recently, it has been demonstrated that OI bone has an increased mineralization kinetics at all developmental stages compared to healthy controls, meaning that the increased mineral content is already present at the onset of mineralization [297].

Hypermineralization associated with a peculiar high bone mass phenotype occurs in the presence of BMP1 mutations or mutations in the BMP1 cleavage site at the C-terminal of procollagen I [74,136]. In both cases, BMP1 fails to remove the C-terminal propeptide, which causes impaired collagen processing in the ECM and abnormal organization of the collagen fibrils [139,140,298].

Another shared bone characteristic among several OI forms is the presence of a high number of osteocyte lacunae that increases bone microporosity, another factor that contributes to fragility at multiple levels [299–301]. The osteocyte lacunar-canalicular network is fundamental for bone homeostasis through signaling between osteoblasts and osteoclasts, but also in the mineralization process, in the degradation and modification of the surrounding matrix composition, in sensing the mechanical stimuli [302–306]. These functions, including dendrite formation, ECM organization, collagen fibril organization, and integrin-mediated signaling pathways, were found dysregulated in OI mouse models [307] and likely contribute to the severity of the phenotype via an abnormal collagen-mineral-matrix to osteocyte interaction [308]. Moreover, osteocytes are known to express OI-related genes, and in OI conditions, osteocytes are found with a highly dysregulated transcriptome that contributes to the OI pathogenesis [307,309]. In particular, osteocytes critically contribute to bone mass by directing osteoclastogenesis through the production of receptor activator of NF-κB ligand (RANKL) and osteoblastogenesis through the secretion of sclerostin, a specific inhibitor of the WNT-signaling pathway [303]. In the context of the cellular imbalance between osteoblasts, osteocytes, and osteoclasts, OI-causing mutations contribute to dysregulation of several signaling pathways important for osteoblast differentiation, bone formation, and bone homeostasis. At first, mutations in WNT1 lead to a reduced capacity to activate WNT signaling pathway, thus affecting osteoblast differentiation as described above [reviewed by Etich et al., 2020 [271]. Also, the RANKL/RANK/NFκβ pathway, despite mutations in its genes that have not been linked to OI, appears to be malfunctioning in the dominant OI mice models *Brtl* and *oim* [273,310]. Particularly, the RANKL/RANK/NFκβ pathway maintains the balance between osteoclasts and osteoblasts, thus regulating bone remodeling. RANKL, mainly secreted by osteocytes, triggers osteoclastogenesis and activates bone resorption on mature osteoclasts [311–313]. However, recent findings demonstrated that RANKL signaling also regulates osteoblastogenesis [314–316] that further exacerbates the bone phenotype observed in OI.

Lastly, perturbations in the TGF-β have important implications in the OI pathogenesis, as TGF-β has a role in bone development and homeostasis [reviewed by Etich et al., 2020 [271]]. Impairment of the TGF-β pathway has been demonstrated in several OI mouse models, with dominant and recessive OI forms that showed increased expression of the TGF-β target genes in bone tissue [93,294,307,317–319]. The mechanisms by which TGF-β signaling is altered in OI remain to be fully understood, but likely ECM signaling may have a role, as changes in the ECM could modify the ECM-associated growth factors, including members of the TGF-β superfamily.

## Targeting Osteogenesis Imperfecta

Clinical management of OI includes physical rehabilitation, surgical correction of bone deformities and fractures, alongside the administration of pharmacological agents with anti-resorptive or anabolic effects on collagen I [320]. Therapeutic strategies target both the intracellular pathways involved in collagen synthesis and the extracellular collagen matrix quantity, addressing the multifaceted pathophysiology of OI. The main challenge in the treatment of OI remains the need to increase bone mass, but also improving bone quality.

### Treatments targeting intracellular pathways

The more recently identified dysregulations of signaling pathways that contribute to the OI pathophysiology offer a broad spectrum of molecular targets for pharmacological intervention. Some of these treatments are currently being tested in clinical trials, while others remain in the preclinical stage waiting for further investigation.

The WNT signaling pathway has been identified as one of the most relevant intracellular targets for treating OI, due to its role in promoting osteoblast activity and bone formation. In particular, research has focused on inhibiting the natural WNT antagonist sclerostin [321–325]. Two monoclonal anti-sclerostin antibodies, romosumab and setrusumab, are currently being evaluated in clinical trials, with their efficacy being compared to standard bisphosphonate treatment.

Another anabolic therapy is based on inhibiting TGF-β. TGF-β signaling is crucial in maintaining bone homeostasis by coupling osteoblast and osteoclast activity. In OI, mutations in collagen I and alterations of the ECM lead to abnormal release and activation of TGF-β, resulting in enhanced TGF-β signaling that contributes to impaired bone formation and increased bone fragility [93,326]. Currently, a clinical trial (MOI-A) is testing whether Losartan, already approved as a drug pressure modulator, is effective in reducing circulating levels of C-terminal telopeptide of type I collagen (CTX), a bone resorption marker, by reducing circulating levels of TGF-β and hence TGF-β pathway signaling.

Treatment of adult patients also occurs via administration of teriparatide (TPTD), an analogue of recombinant human parathyroid hormone (PTH). The clinical trial testing TPTD showed increased bone formation and bone resorption markers in OI patients, although the effects were more pronounced in the milder OI forms [327,328]. Nowadays, the clinical trial TOPaZ is testing TPDP treatment followed by a single-dose administration of zoledronate, an antiresorptive drug, to understand whether the drug combination has beneficial effects on reducing the fracture risk in adult OI patients [329].

Emerging innovative treatment options are under investigation, aiming at restoring cellular homeostasis, demonstrated to be altered in several OI forms. In this context, recent studies on OI patient fibroblasts and OI animal models aiming at targeting ER stress and UPR pathway have shown that the administration of the chemical chaperone 4-phenylbutyrate (4-PBA), as well as a more stable modified version of this molecule, namely N-benzylglycine, improved cell homeostasis [54,61,98,330,331]. More recently, an *in vitro* study using human OI fibroblasts demonstrated that administration of exogenous HSP47 restores intracellular homeostasis by facilitating proper collagen folding and secretion. This led to improved assembly and incorporation of structurally more stable collagen into the ECM, ultimately enhancing matrix organization and function [332]. These findings highlight the therapeutic potential of targeting molecular chaperones like HSP47 to specifically correct collagen misfolding.

It should be noted that the pathophysiology and molecular mechanisms underlying OI vary significantly across the different forms. This molecular heterogeneity makes it essential to identify and target the specific pathways altered in each patient. Consequently, grouping OI patients based on their defective molecular mechanisms is crucial to enable effective patient stratification [32].

### Treatments targeting the extracellular environment

The most widely used drugs in OI consist of the anti-osteoporotic bisphosphonates that decrease bone resorption, thus diminishing bone remodeling by inducing osteoclast apoptosis [333] overall improving bone mass. Nevertheless, these drugs have notable limitations. Specifically, bisphosphonates do not correct the primary molecular defect caused by mutations in collagen I, thus improving bone quantity, but not its quality. Their efficacy in fracture reduction is variable and generally less pronounced in adults, likely due to decreased osteoblastic activity [334,335]. Furthermore, long-term use may be associated with side effects such as a low risk of osteonecrosis of the jaw and potential accumulation of microfractures [334,336].

Another available treatment targeting the extracellular environment is Denosumab, a monoclonal antibody against RANKL. Denosumab, currently approved for the treatment of primary osteoporosis [337], reduces osteoclast differentiation, activity, and survival [320] inhibiting ECM remodeling; however, about half of the OI-affected children treated developed serious hypercalcemic crisis as a rebound effect after discontinuation of the treatment. For this reason, Denosumab should not be used as first-line therapy in children with OI [338,339]. In adult patients, instead, Denosumab seems to be a promising option as it could increase bone mass [340]. Further studies are still needed, especially to elucidate potential side effects [320,338–340].

Recently, dietary approaches have been used in animal models to target the hypermineralization typical of OI bone. In particular, a low dietary phosphate approach gained beneficial effects in ameliorating the skeletal defects and in reducing the hypermineralization phenotype in a zebrafish model of dominant OI [341,342].

## Conclusion

Which is the main molecular determinant responsible for OI severity between abnormal bone matrix and altered intracellular homeostasis? Can we associate the alteration of collagen I structure and/or its intracellular accumulation and/or poor secretion to the degree of severity? In OI, defective collagen structure or alterations in proteins involved in collagen biosynthesis or bone cell activity play a crucial role in the pathophysiology of the disease. In OI forms characterized by over- or under-modified collagen I, its intracellular accumulation and poor secretion and/or incorporation represent a shared hallmark, and generally, the clinical outcome is more severe. Interestingly, quantitative defects associated with collagen I haploinsufficiency cause mild outcomes, whereas the synthesis of normally post-translationally modified collagen I in other OI forms results in moderate to severe and even lethal outcomes. This appears true independently of collagen intracellular accumulation supporting a crucial role of the overall cell homeostasis in modulating disease severity. The combined effect of extracellular and intracellular consequences of aberrant mutant collagen synthesis must be taken into account to fully understand OI severity. Targeting aberrant ECM may be challenging, thus the identification of molecular cellular targets within the collagen I biosynthetic pathways and/or bone-forming cell differentiation appears to be crucial for the development of innovative OI therapies. By restoring cellular homeostasis, it may be possible to improve bone mass and quality, with the ultimate goal to ameliorate patient health.

While animal models have been and are invaluable tools for understanding OI pathogenesis, notable discrepancies exist between these models and human patients. Animal models often exhibit more uniform phenotypes, whereas human OI shows broad clinical variability influenced by diverse genetic backgrounds and modifier genes. Not always does animal severity recapitulate the human one. Differences in bone structure, remodeling rates, and molecular pathways further limit direct extrapolation of findings. Additionally, therapeutic responses in animals do not always predict clinical outcomes in humans. These discrepancies underscore the need to integrate insights from both models and patient-derived data to fully elucidate OI mechanisms and improve treatment strategies.

Looking ahead, emerging research into epigenetic regulation may uncover novel mechanisms influencing OI phenotypes and offer new therapeutic targets. Furthermore, advances in precision gene-editing technologies such as CRISPR/Cas systems present exciting opportunities to correct pathogenic mutations directly, potentially transforming the treatment landscape for OI. Despite these advances, unanswered questions remain regarding the precise molecular pathways linking collagen defects to bone cell dysfunction, the long-term safety of gene editing approaches, and how best to integrate multi-omic data to predict disease severity and treatment responses. Addressing these gaps will be critical to translate molecular insights into effective, personalized therapies for OI.

**Clinical Perspective**

- Our understanding of OI has undergone significant advancements over the past two decades. It is now evident that OI is not merely a disease of collagen I but represents a broader group of collagen I-related conditions. These disorders arise not only from mutations in the genes encoding collagen I, but also from defects in various proteins and enzymes involved in critical steps of collagen synthesis, as well as in the processes regulating bone-forming cell differentiation and activity.
- Furthermore, new literature data revealed that OI is affecting both the extracellular matrix as well as the homeostasis of collagen I producing cells.
- Thus, it becomes relevant to have a deep overview of all the so far identified 23 OI and 5 OI-like forms, to identify new targets for innovative therapeutic approaches aiming at enhancing bone mass and improving bone quality in OI patients.

## Data Availability

This is a review article with no primary data, so not applicable.

## Open Access

This article has been published open access under our Subscribe to Open programme, made possible through the support of our subscribing institutions, learn more here: https://portlandpress.com/pages/open_access_options_and_prices#conditional

## Abbreviations

ADAM2: ADAM metallopeptidase domain 2
AFM: atomic force microscopy
ALPL: alkaline phosphatase
ATF4: activating transcription factor 4
ATF6: activating transcription factor 6
Akt: protein kinase B
BMD: bone mineral density
BMP1: bone morphogenic protein 1
BMP2: bone morphogenic protein 2
BRIL: bone-restricted interferon-induced transmembrane protein-like
BV/TV: bone volume/tissue volume
Bglap2: bone gamma-carboxyglutamate protein 2
BiP: immunoglobulin heavy-chain-binding protein
CCDC134: coiled-coil domain-containing protein 134
CNP: C-type natriuretic peptide
COL1A1: collagen 1 alpha 1
COL1A2: collagen 1 alpha 2
COPI: coat protein complex I
COPII: coat protein complex II
CREB/ATF: AMP response element-binding protein/activating transcription factor
CREB3L1: cAMP responsive element binding protein 3-like 1
CRISPR/Cas: clustered regularly interspaced palindromic repeats/ CRISPR-associated proteins
CRTAP: cartilage associated protein
CTX: C-terminal telopeptide of type I collagen
CaMKII: Ca^2+^ calmodulin kinase II
CypB: cyclophilin B
DI: dentinogenesis imperfecta
DI: dentinogenesis imperfecta
DICKKOPF: dickkopf-related protein 1
ECM: extracellular matrix
EDS: Ehlers-Danlos syndrome
ER: endoplasmic reticulum
ERAD: ER-associated degradation
ERK: extracellular signal-regulated kinase
FAM46A: family with sequence similarity 46, member A
FKBP10: FK506 binding protein 10
FKBP65: 65 kDa FK506 binding protein
HP/LP: hydroxylysylpyridinoline/ lysylpyridinoline
HSP47: heat shock protein 47
HSP70: heat shock protein 70
Hyl: hydroxylysine
Hyp: hydroxyproline
IBSP: integrin-binding sialoprotein
IFITM5: interferon-induced transmembrane protein 5
IP3R: inositol 1,4,5-triphosphate receptor
IRE1-XBP1: inositol-requiring enzyme 1- X-box binding protein 1
ISR: integrated stress response
KDEL: Lys-Asp-Glu-Leu
KDELR2: KDEL receptor 2
LEF: lymphoid enhancer-binding factor
LH1: lysyl hydroxylase 1
LH2: lysyl hydroxylase 2
LH3: lysyl hydroxylase 3
LOX: lysyl oxidase
LRP5/6: frizzled/low density lipoprotein receptor-related protein 5 or 6
MAPK: mitogen-activated protein kinase
MBTPS2: membrane-bound transcription factor peptidase site 2
MESD: mesoderm development candidate 2
MLBR: major ligand binding regions
NF-κB: nuclear factor kappa-light-chain-enhancer of activated B cells
NMD: nonsense-mediated decay
OASIS: old astrocyte specifically induced substance
OI: osteogenesis imperfecta
4-PBA: 4-phenylbutyrate
PDI: protein disulfide isomerase
PEDF: pigment epithelium-derived factor
PERK: protein kinase R-like endoplasmic reticulum kinase
PERK: protein kinase R-like endoplasmic reticulum kinase
P3H1: prolyl 3-hydroxylase 1
P4H1: prolyl-4-hydroxylase 1
P4HB: prolyl 4-hydroxylase subunit beta
PHLDB1: pleckstrin homology-like domain family B member 1
PKR: protein kinase R
PLOD2: procollagen-lysine,2-oxoglutarate 5-dioxygenase 2
PLS3: plastin-3
PPIB: cyclophilin B
PPIases: peptidyl-prolyl cis-trans isomerases
PTC: premature termination codon
PTH: parathyroid hormone
PTMs: post-translational modifications
RANK: receptor activator of NF-κB
RANKL: receptor activator of NF-κB ligand
RIP: regulated intramembrane proteolysis
ROS: reactive oxygen species
Runx2: runt-related transcription factor 2
SEC24D: sec24 homolog D
SERPINF1: serpin family F member 1
SERPINH1: serpin family H member 1
SMAD: suppressor of mother against decapentaplegic
SOST: sclerostin
SOX9: SRY-box transcription factor 9
S1P: site 1 metalloprotease
S2P: site 2 metalloprotease
SP7: Sp7 transcription factor
SPARC: secreted protein acidic and rich in cysteine
SPP1: osteopontin
TANGO1: transport and Golgi organization 1
TCF: T-cell factor
TENT5A: terminal nucleotidyltransferase 5A
TGFβ: transforming growth factor β
TMEM38B: transmembrane Protein 38, member B
TPTD: teriparatide
TRIC-B: trimeric intracellular cation channel type B
TbN: trabecular number
UPR: unfolded protein response
WNT1: WNT family member 1
WNT: wingless-related integration site
bZIP: basic leucine zipper
mRNA: messenger ribonucleic acid
